# Incidence of Complications Related to Corneal Graft in a Group of 758 Patients

**DOI:** 10.3390/jcm12010220

**Published:** 2022-12-28

**Authors:** Dominika Szkodny, Ewa Wróblewska-Czajka, Adam Wylęgała, Magdalena Nandzik, Edward Wylęgała

**Affiliations:** 1 Chair and Clinical Department of Ophthalmology, Faculty of Medical Sciences, Zabrze Medical University of Silesia in Katowice, 40-760 Katowice, Poland; 2 Department of Ophthalmology, District Railway Hospital in Katowice, 40-760 Katowice, Poland; 3 Health Promotion and Obesity Management, Department of Pathophysiology, Medical University of Silesia, 40-760 Katowice, Poland

**Keywords:** penetrating keratoplasty, corneal graft, corneal perforation, keratitis, regraft, keratoplasty indications, keratoplasty complications

## Abstract

Purpose: this study aimed to assess the frequency of complications related to corneal grafts, including epithelialization disorders, wound dehiscence, infectious keratitis, recurrence of herpetic keratitis, graft rejection, late graft failure, and infectious and noninfectious corneal melting, while also considering risk factors, particularly indications. Methods: this retrospective analysis of corneal graft failure included a chart review of the hospital records of patients who underwent penetrating keratoplasty (PK) between January 2016 and December 2020 at the Department of Ophthalmology of the District Railway Hospital, Katowice, Poland. Results: Between 2016 and 2020, a total of 758 PK procedures were carried out at the ophthalmology department. Bullous keratopathy (20.58%), keratoconus (18.07%), and corneal perforation (13.32%) were the primary indications for keratoplasty. Secondary glaucoma was diagnosed in 99 patients (13.06%). The success rate of PK was 72.43% (494). The most frequent treatment complication was secondary glaucoma (13.06%), followed by late endothelial failure, perforation (4.1%), and bacterial keratitis (3.23%). Patients in the high-risk group were 4.65 times more likely to develop complications than those in the low-risk group. Multivariate regression analysis showed that concomitant ophthalmic diseases (odds ratio (OR): 3.12, confidence interval (CI): 1.60–6.08, *p* = 0.00) and connective tissue diseases (OR: 7.76, CI: 2.40–25.05, *p* = 0.00) were significant factors associated with the occurrence of complications. Diabetes, dermatological diseases, primary glaucoma, and sex were not associated with corneal graft failure (*p* > 0.05). Conclusion: Chronic loss of the endothelium was the primary cause of graft failure in individuals who underwent PK. The high-risk transplant has up to 4.65 times higher risk of complications compared to the indications with a good prognosis.

## 1. Introduction

The need for a corneal transplant can arise for a variety of reasons, such as corneal ectasia, bullous keratopathy, corneal stromal dystrophies, Fuchs’ endothelial dystrophy, scarring, perforation, and corneal graft failure. The frequency of certain indications varies over time and is influenced by the demographics of the patients as well as the socioeconomic background of the country [1]. Despite the great success rate of corneal transplantation, there are still certain risks involved [2]. Some of these are primary graft failure, immunological allograft rejection, late endothelial failure, intraocular pressure elevation, ocular surface diseases, wound dehiscence, graft detachment, keratitis, and keratitis endophthalmitis [2]. To minimize patient morbidity and preserve the transplant, early diagnosis and prompt treatment is crucial. Any structural disorder in the corneal button that disturbs vision can be referred to as graft failure. This condition may arise from many distinct pathways [3]. The risk factors for graft failure include initial diagnosis, ocular surface diseases, atopy, connective tissue diseases, previous herpes keratitis, patient age, graft duration, corneal vascularization, suture-related problems, and previous graft failure [4]. Due to the high rate of immune-mediated graft rejection and graft neovascularization, high-risk keratoplasty considerably lowers the rate of transplant success [5]. The success rate of penetrating keratoplasty (PK) has been shown to range from 52% to 98.8% [2].

Knowledge of risk factors and their influence on transplant survival allows undertaking various preoperative, intraoperative, and postoperative preventive measures and also helps to improve patient prognosis for graft success. This study assessed the frequency of complications related to corneal graft, considered the risk factors, especially indications, which are the primary determinants of transplant success, and attempted to provide updated information on this topic.

## 2. Patients and Methods

This retrospective, observational study included a chart review of the hospital records of patients who underwent PK between January 2016 and December 2020 at the Department of Ophthalmology of the District Railway Hospital in Katowice, Poland. The study adhered to the tenets of the Declaration of Helsinki. The data collected for the study included patients’ age and sex, date of surgery, reason for surgery, corneal transplant technique, preoperative visual acuity, concomitant conditions, eye surface disorders, number of previous corneal transplantations, lens status, presence of glaucoma before and after keratoplasty, postoperative visual acuity, occurrence of any complications following the surgery, and any surgical interventions after keratoplasty. The complications found in the analyzed group were classified as follows: epithelialization disorders, wound dehiscence, infectious keratitis, recurrence of herpetic keratitis, graft rejection, secondary glaucoma, late graft failure, and infectious and noninfectious corneal melting. The incidence of these complications in the “low-risk” and “high-risk” graft failure groups was compared. Patients with conditions such as ocular burn and limbal stem cell deficiency, perforation, keratitis, at least one graft decompensation, or a history of herpetic keratitis were considered to be at high risk. In addition, the number of unique complications, depending on the indications and other risk factors such as connective tissue diseases, accompanying eye diseases, and previous corneal transplants, and the odds ratio (OR) of complication occurrence in low- and high-risk transplants were examined. Data are presented as the number of cases with a percentage value. The relationships between the qualitative variables were compared using the Pearson chi^2^ test. The influence of the studied factors on the occurrence of complications was assessed by generating a single and multifactor logistic regression model with selected risk factors included. Data for the model are presented as ORs with 95% confidence intervals (CIs). *p* values < 0.05 were considered significant. Statistical analysis was performed using the RStudio software in the R language.

## 3. Results

A total of 758 PK procedures were carried out at the Ophthalmology Department of the District Railway Hospital in Katowice between January 2016 and December 2020. Of these patients, 76 skipped more than one follow-up. The main indications for keratoplasty were bullous keratopathy (20.58%), keratoconus (18.07%), corneal perforation (13.32%), Fuchs’ dystrophy (12.27%), graft failure (10.16%), dystrophies other than Fuchs’ (7.39%), traumatic scar of the cornea (5.01%), bacterial keratitis (3.17%), fungal keratitis (1.72%), herpetic keratitis (1.19%), chemical burn of the cornea (0.53%), and acanthamoeba keratitis (0.26%) (Table 1).

The primary procedures were performed in 557 (73.48%) patients, the second corneal transplant in 127 (16.75%), the third in 19 (2.5%), the fourth keratoplasty in 7 (0.92%), the fifth in 1 (0.13%), and the sixth also in 1 (0.13%). Primary glaucoma was diagnosed in 170 patients (22.42%), while secondary glaucoma was noted in 99 patients (13.06%). Before the keratoplasty, 398 (52.24%) patients were phakic, 291 (38.39%) were pseudophakic, and 22 (2.9%) were aphakic. The majority of patients (90.24%) did not have any comorbid ophthalmic conditions; the rest had had at least one of the following: a history of uveitis, prior herpetic keratitis, chemical burn, eyelid disorders, ocular synechia, multiple vitrectomies, dry eye syndrome, limbal stem cell deficiency, or bullous pemphigoid. Thirty (3.96%) patients had suffered from diabetes, 23 (3.02%) from connective tissue disease, and 5 (0.65%) from dermatological diseases (Table 2).

**Figure 1:**
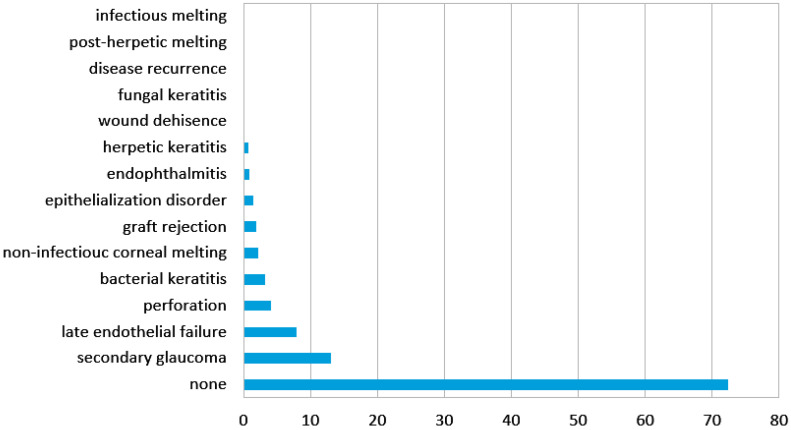
Frequency of corneal graft complications in the analyzed group.

The complications related to corneal graft are listed in Figure 1. The most common ones were secondary glaucoma (13.06%), late endothelial failure (7.92%), perforation (4.1%), bacterial keratitis (3.23%), noninfectious corneal melting (2.2%), graft rejection (1.9%), epithelialization disorders (1.47%), endophthalmitis (0.88%), herpetic keratitis (0.73%), wound dehiscence, fungal keratitis, primary disease recurrence, and postherpetic melting and infectious melting (0.15%).

**Figure 2:**
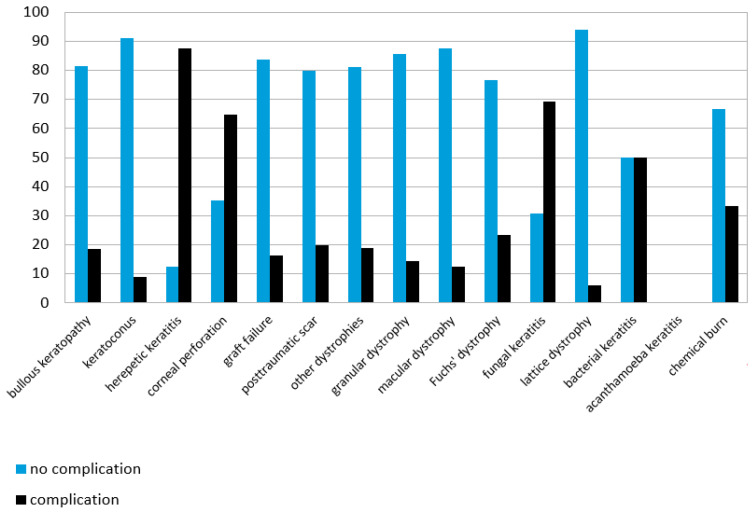
Success rate of penetrating keratoplasty depending on the indication.

The failure rate of PK depended on the complications (Figure 2).

Patients with acanthamoeba keratitis (0%), herpetic keratitis (12.5%), fungal keratitis (30.8%), or perforation (35.2%) had the lowest success rate, while those with lattice corneal dystrophy (94.1%), keratoconus (91.0%), macular corneal dystrophy (87.5%), granular corneal dystrophy (85.7%), and graft decompensation (83.8%) had the highest success rate (Figure 2).

Following PK, 85 (12.46%) patients required another ophthalmic surgery, including corneal regraft (8.2%), amniotic membrane transplant (2.05%), evisceration (1.76%), and wound suturing (0.15%). Patients in the high-risk group were 4.65 times more likely to develop complications than those in the low-risk group. Previous corneal graft failure (*p* = 0.00), indication for keratoplasty (*p* = 0.00), concomitant ophthalmic diseases (*p* = 0.00), and connective tissue diseases (*p* = 0.00) were significantly associated with an increased risk of graft failure. On the other hand, diabetes, dermatological diseases, primary glaucoma, and sex were not linked with the occurrence of complications (*p* > 0.05).

**Figure 3:**
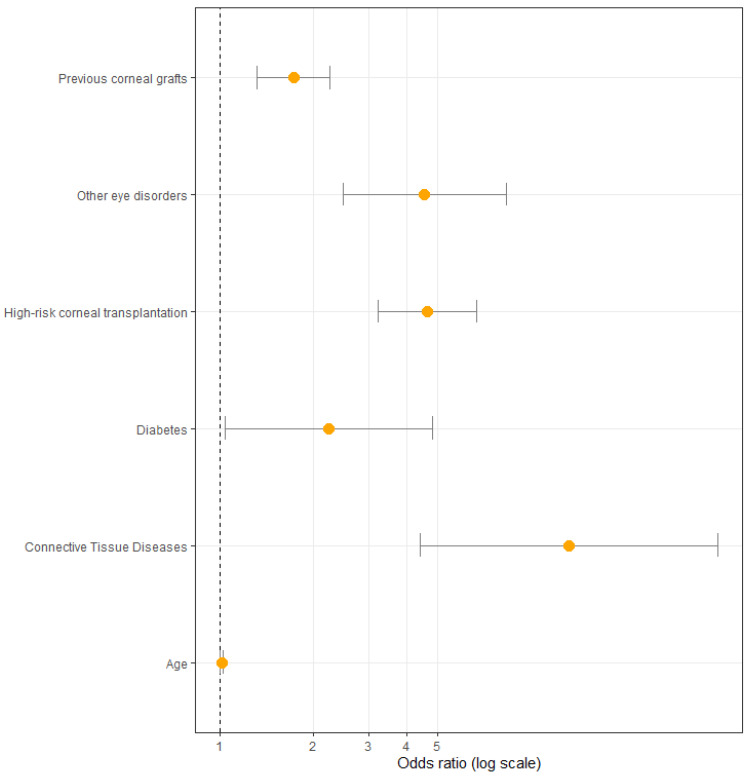
Odds ratio with confidence intervals of the studied factors included in univariate analysis.

Several factors were included in the univariable analysis (Figure 3). The following were the factors associated with graft failure: age (OR: 1.01, CI: 1.00–1.02, *p* = 0.03), high-risk group (OR: 4.65, CI: 3.22–6.72, *p* = 0.00), previous corneal transplants (OR: 1.73, CI: 1.32–2.26, *p* = 0.00), concomitant ophthalmic diseases (OR: 4.56, CI: 2.49–8.33, *p* = 0.00), connective tissue diseases (OR: 13.29, CI: 4.40–40.08, *p* = 0.00), and diabetes (OR: 2.23, CI: 1.03–4.82, *p* = 0.04).

**Figure 4:**
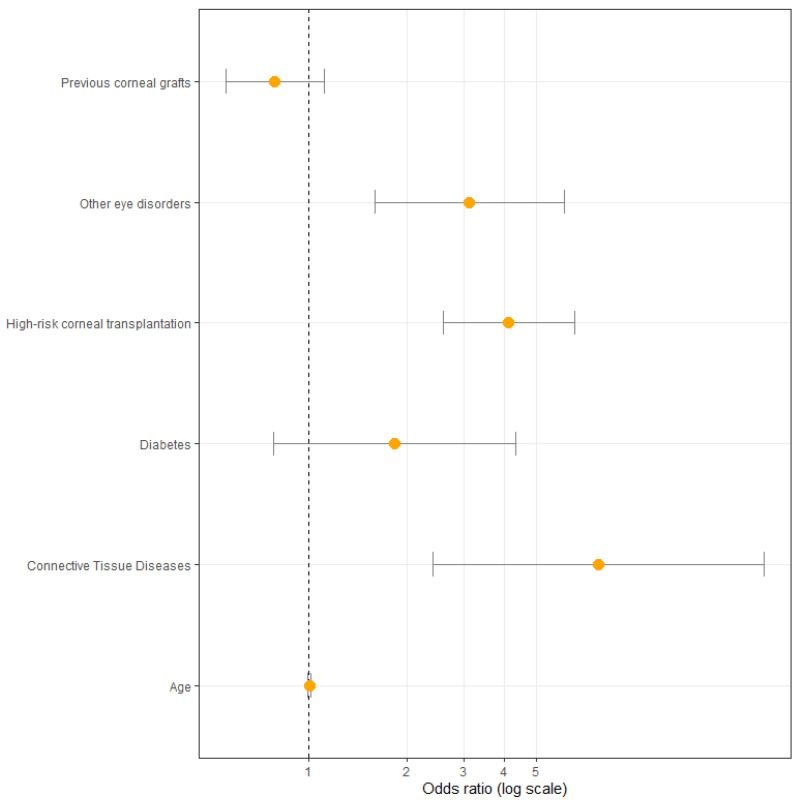
Odds ratio with confidence intervals of the studied factors included in multivariate analysis.

Multivariate regression analysis (Figure 4) revealed three factors to be significantly associated with complication occurrence. These include high-risk group (OR: 4.12, CI: 2.59–6.55, *p* = 0.00), concomitant ophthalmic diseases (OR: 3.12, CI: 1.60–6.08, *p* = 0.00), and connective tissue diseases (OR: 7.76, CI: 2.40–25.05, *p* = 0.00).

## 4. Discussion

Despite the increasing success of lamellar keratoplasties, PK is widely used in many countries, including Poland [6]. The main reason for this trend is the shortage of corneal tissue for transplantation [7]. Our study assessed the success rate and frequency of complications after PK in the District Railway Hospital in Katowice, which is one of the major tertiary eye-care centers in the country. The presented results will contribute to expanding the knowledge on the outcome of PK performed in Poland as there are insufficient studies on this topic in this nation compared to other European countries. This study included heterogenous corneal disorders as a significant number of high-risk patients require unique care. Complications associated with each indication were further assessed separately for a more precise comparison to the existing literature.

Our study showed that the success rate of keratoplasty was 72.43%, which is comparable to other reports [1,8]. The most common disorders related to corneal button were found to be endothelial failure, perforation, and bacterial keratitis, which significantly contradicts those reported in the study by M. Muraine et al. [9]. This finding also differs from the results of most of the other previous studies showing a higher number of graft rejections [1,10,11,12]. Nevertheless, some reports have shown late endothelial failure as the leading cause of graft failure, which is similar to our result [13,14].

It is highly likely that among patients with endothelial failure were those who have had a graft rejection but did not show up quickly enough to diagnose it. Corneal graft rejection and inflammation are significant factors of endothelial graft failure [15]. Severe rejection has been proven to be reversible in up to two-thirds of the cases, with graft transparency being maintained in two-thirds of them [14]. In our analysis, the incidence of graft rejection was found to be low (1.9%), which suggests that it is underdiagnosed. The likelihood of endothelial decompensation after rejection increases with the duration between corneal graft rejection and treatment [16]. Therefore, it is important to educate patients about the symptoms of graft rejection and the need to immediately report to the hospital.

Surprisingly, perforation was found to be the second most common complication after PK in the study period. This fact should be explained in the framework of indications in the study group, with perforation being the third most common complication. Because our hospital is a tertiary referral hospital where *most corneal transplants are performed in our region*, a lot of patients with severe corneal conditions are referred to us, and the incidence of perforation is significant. Furthermore, it is well known that corneal transplantation has a low success rate [1]. Based on our results, it can be concluded that patients are not diagnosed and referred to our hospital early enough to prevent perforation and improve the likelihood of a successful transplant.

In our study, the incidence of infectious keratitis was 3.23%, which is within the range of this complication being reported in developed countries (1.5–11.6%) [4,17,18]. In addition, corneal perforation was identified to be the most frequent indication for keratoplasty resulting in microbial keratitis. Due to the retrospective nature of our study and lack of knowledge of the etiology of corneal perforation, we can only speculate that many of these cases also started with keratitis [19]. Because failed graft is a long-term risk factor for graft infection, perforation, regardless of its cause, can more likely lead to keratitis [20]. 

The study shows that fungal keratitis, herpetic keratitis, acanthamoeba keratitis, and perforation are associated with the worst prognosis after keratoplasty [18], which corresponds to low survival rates of therapeutic keratoplasty described in other studies [1]. The total success rate of PK is around 90%, which is much higher compared to high-risk transplants rarely reaching 40% [21]. However, given the limited number of procedures performed on the analyzed group, the exact success rate should be further investigated. It has been reported that early intervention can help to improve outcomes [22], so timely diagnosis is crucial for the success of corneal transplantation.

Another intriguing finding was that as many as 45% of patients with complications related to corneal button needed surgical intervention, of which the vast majority (29.79%) required regraft. In addition, the primary indication for almost half of the patients who required retransplantation was perforation (44.64%).

According to some reports, transplant failure is determined by both donor and recipient factors. The indication, prior corneal transplants, neovascularization, prior graft rejection, and type of transplant are known to influence the graft outcome [23]. Additionally, donor variables such as endothelial cell density, donor age, and H-Y incompatibility can play a role in graft rejection and failure. Some donor characteristics, such as race, lens status, and harvesting and preservation techniques and recipient factors, such as diabetes, have been studied so far, but the reported results are often conflicting [15].

Known risk factors such as previous corneal graft, indication, other ophthalmic diseases, and connective tissue diseases were found to be significantly associated with an increased risk of graft failure [24].

Although diabetes and sex are controversial risk factors for graft failure [3], glaucoma is an established cause [25,26], As described in a recent review, glaucoma is the main cause of vision loss after PK and also of graft failure [27,28]. Treatment with steroids has been shown to cause post-PK glaucoma; however, changes in the iridocorneal angle, resulting from collapse and distortion of the trabecular meshwork, may also contribute to intraocular pressure elevation [29]. Furthermore, pre-existing glaucoma is another risk factor for the development of secondary glaucoma and may lead to increased loss of corneal endothelial cells and irreversible optic nerve damage. The use of the antimetabolites mitomycin C and 5-fluorouracil, which are toxic to the endothelium, can lead to graft failure [30]. Ing et al. reported that post-PK glaucoma can decrease graft survival as graft survival was found in only 58% of patients with pre-existing glaucoma compared to 80% of patients without glaucoma in a 10-year follow-up [29]. Nevertheless, our study did not show such an association between glaucoma and graft survival. This finding should be interpreted with caution because retrospective studies may be characterized by a lack of data. Moreover, the observation time was relatively short compared to other studies and the endpoints studied were complications related to corneal button, while best corrected visual acuity and effect of glaucoma on vision loss were not assessed. In our study, we noted that antimetabolites were not used for glaucoma surgeries which cause endothelial cell loss. It should be mentioned that diagnosis and monitoring of PK-associated glaucoma is not always simple due to the lower reliability of intraocular pressure measurements after a corneal graft.

Neovascularization was not examined in our study, although it is another recognized factor that is known to cause graft failure and graft rejection [21,31,32]. This is due to insufficient data which prevented the assessment of this factor. However, not all studies showed neovascularization as an independent risk factor as it is associated with other factors, such as indication [31].

Assessment of the correlation between many variables and the occurrence of graft complications using the multivariate analysis led to the identification of three important variables: a high-risk group, concomitant ophthalmic diseases, and connective tissue diseases. As the high-risk group represents patients with specific indications, it can be assumed that the indication for corneal transplantation is a significant risk factor, as reported in several previous studies [15,21,22,33,34,35,36].

## 5. Conclusions

In summary, one of the main causes of graft failure is chronic loss of the endothelium after PK. The occurrence of complications is mainly dependent on the indication for corneal transplantation, and, even after years, it remains a major treatment challenge.

The high-risk transplant is up to 4.65 times more likely to develop complications than the indications with a good prognosis. It is essential to take efforts to improve the chances of graft survival, including advancements in corneal preservation methods, surgical techniques, and the use of more potent antirejection drugs. However, it appears that a key strategy is to ensure appropriate patient follow-up and educate patients to identify severe corneal conditions at an earlier stage.

## Figures and Tables

**Table 1:** Indications for keratoplasty in the studied group.

Indication	Frequency (%)
Bullous keratopathy	20.58
Keratoconus	18.07
Corneal perforation	13.32
Fuchs’ dystrophy	12.27
Graft failure	10.16
Other dystrophies	7.39
Traumatic scar	5.01
Bacterial keratitis	3.17
Fungal keratitis	1.72
Herpetic keratitis	1.19
Chemical burn	0.53
Acanthamoeba keratitis	0.26

**Table 2:** Patient characteristics.

Previous corneal transplants	Frequency (%)
None	73.48
1	16.75
2	2.5
3	0.92
4	0.13
5	
Primary glaucoma	
Yes	22.42
No	77.58
Lens status	
Phakic	52.24
Pseudophakic	38.39
Aphakic	2.9
Other ophthalmic diseases	
Yes	9.76
No	90.24
Diabetes	
Yes	3.96
No	96.04
Connective tissue disease	
Yes	96.98
No	3.02
Dermatological disease	
Yes	0.65
No	99.35

The success rate of PK in the study group was 72.43% (494).

## Data Availability

Data are available on request due to restrictions (e.g., privacy or ethical).
